# Miller Fisher syndrome after COVID-19 vaccination: Case report and review of literature

**DOI:** 10.1097/MD.0000000000029333

**Published:** 2022-05-27

**Authors:** Ahsun Rizwan Siddiqi, Tehrim Khan, Muhammad Junaid Tahir, Muhammad Sohaib Asghar, Md. Saiful Islam, Zohaib Yousaf

**Affiliations:** a Wah Medical College, Affiliated with University of Health Sciences, Wah, Pakistan; b Lahore General Hospital, Lahore, Pakistan; c Dow University of Health Sciences–Ojha Campus, Karachi, Pakistan; d Department of Public Health and Informatics, Jahangirnagar University, Savar, Dhaka-1342, Bangladesh; e Centre for Advanced Research Excellence in Public Health, Savar, Dhaka-1342, Bangladesh; f Hamad Medical Corporation, Doha, Qatar.

**Keywords:** case report, COVID-19, Guillain-Barre syndrome, Miller Fisher syndrome, vaccinations

## Abstract

**Rationale:**

Miller Fisher syndrome (MFS) is a rare variant of Guillain-Barre syndrome, classically diagnosed based on the clinical triad of ataxia, areflexia, and ophthalmoplegia. MFS is usually preceded by viral infections and febrile illness; however, only a few cases have been reported after vaccinations.

**Patient concerns:**

A 53-year-old hypertensive male presented with a 2-day history of progressive ascending paralysis of the lower limbs along with diplopia and ataxia, 8 days after the first dose of the Sinovac–Coronavac coronavirus disease 2019 (COVID-19) vaccination, with no prior history of any predisposing infections or triggers.

**Diagnoses:**

Physical examination showed moderate motor and sensory loss with areflexia in the lower limbs bilaterally. Routine blood investigations and radiological investigations were unremarkable. Cerebrospinal fluid analysis showed albuminocytologic dissociation and nerve conduction studies revealed prolonged latencies with reduced conduction velocities. The diagnosis of MFS was established based on the findings of physical examination, cerebrospinal fluid analysis, and nerve conduction studies.

**Interventions:**

A management plan was devised based on intravenous immunoglobulins, pregabalin, and physiotherapy. However, due to certain socioeconomic factors, the patient was managed conservatively with regular physiotherapy sessions.

**Outcomes:**

Follow-up after 6 weeks showed remarkable improvement, with complete resolution of symptoms 10 weeks after the discharge.

**Lessons:**

This case suggests that MFS is a rare adverse effect after COVID-19 vaccination and additional research is required to substantiate a temporal association. Further studies are needed to understand the pathophysiology behind such complications to enhance the safety of COVID-19 vaccinations in the future.

## 1 Introduction

Miller Fisher syndrome (MFS) is a rare variant of Guillain-Barre syndrome (GBS).^[1]^ Typical presentation of GBS is a symmetric, progressive, ascending paralysis with associated sensory deficits and impaired reflexes. MFS classically presents as a clinical triad of ataxia, areflexia, and ophthalmoplegia.^[1,2]^ MFS is a relatively uncommon acute autoimmune neuropathy, accounting for only 5% of patients with GBS.^[3]^ MFS is frequently (84%) preceded by viral infections of the gastrointestinal or respiratory tracts. *Campylobacter jejuni* is the commonest triggering infection worldwide.^[4]^ Bickerstaff brainstem encephalitis (BBE) is a condition similar to MFS with a triad of acute bilateral ophthalmoplegia, ataxia, and encephalitis with an overlap with other GBS variants.^[5]^

There is ambiguous data on the association of vaccinations with autoimmune neuropathies, most notably the incidence of GBS following influenza vaccination.^[6]^ With the widespread and accelerated coronavirus disease 2019 (COVID-19) immunization programs, cases of postvaccination-GBS are increasingly being reported.^[7]^ However, relatively fewer cases of MFS have been reported after COVID-19 vaccination. Here, we present the first presumptive case of MFS in the South East Asian region following the first dose of the inactivated COVID-19 vaccine.

## 2 Case presentation

A 53-year-old Asian (Pakistani) gentleman, known hypertensive with ischemic heart disease for the last 6 years, noncompliant with his medications, and a chronic smoker for the past 30 years, presented with a 2-day history of sudden onset of progressive weakness of the bilateral lower limbs. The weakness was symmetrical and ascending, with difficulty in walking and mild weakness of the upper limbs bilaterally. These symptoms were associated with paresthesia and tingling, along with intense myalgias in the lower limbs. The patient also complained of dribbling of saliva and inability to fully close the right eye. The patient received the first dose of the Sinovac-CoronaVac COVID-19 vaccine 8 days prior to the onset of symptoms. There was no previous history of any respiratory or gastrointestinal illness. There was no history of any febrile illness that preceded or coincided with the muscle weakness. Also, there was no evidence of any other predisposing infectious or autoimmune disorders. A complete review of systems was unremarkable.

Upon presentation, the patient was well oriented in time, place, and person. He was afebrile (36.7°C), normotensive (110/70 mm Hg), not tachycardiac (83/min), not tachypneic (17/min), and was maintaining oxygen saturation of 98% at room air.

On physical examination, the power was 3/5 in both the lower limbs with the ability to overcome gravity but an inability to move the limb against resistance. These findings were predominantly present in the plantar flexor, dorsiflexor, and quadricep muscle groups. There was no atrophy or wasting of the lower limb muscles. Deep tendon reflexes were absent in the lower limbs bilaterally. Power was 4/5 in the upper limbs bilaterally. The rest of the motor examination was normal. Pain, touch, and vibration sensations were intact but there was a mildly reduced sense of proprioception in both the lower limbs. The patient had an ataxic gait and the Romberg sign was positive. The rest of the lower limb cerebellar examination was unremarkable. The upper limb had intact deep tendon reflexes, and the sensory and cerebellar examinations were unremarkable. Cranial nerve examination revealed horizontal gaze diplopia with an inability to abduct the right eye. He was diagnosed with ophthalmoplegia due to right-sided Abducens nerve (CN VI) palsy. The facial signs were consistent with right-sided lower motor neuron facial palsy; however, the corneal reflex was intact. The rest of the cranial nerve examination was unremarkable. Meningeal signs were absent. The rest of the examination, including a detailed chest and abdominal examination, was normal.

Investigations, including complete blood count, renal and liver function tests, were normal. (Table 1) A noncontrast computed tomography scan of the brain ruled out any acute hemorrhage, after which he received aspirin 300 mg and clopidogrel 300 mg, considering the possibility of acute ischemic stroke. The brain's magnetic resonance imaging showed no abnormalities, and antiplatelets were discontinued. Cerebrospinal fluid (CSF) analysis showed elevated proteins in the CSF without pleocytosis. Nerve conduction studies and electromyography performed 10 days after the onset of symptoms showed prolonged latencies with reduced conduction velocities and prolonged F waves delineating acute inflammatory demyelinating polyneuropathy (Table S1, Supplemental Digital Content). Diagnostic testing for *C jejuni* was not performed due to the absence of gastrointestinal symptoms. Anti-GQ1b antibody test along with confirmatory tests for viral agents were not performed in our patient due to the lack of availability in our region. The absence of bilateral symptoms and encephalitis made any alternative diagnosis including Bickerstaff brainstem encephalitis unlikely. The patient was diagnosed as a case of MFS based on the clinical manifestations of ataxia, ophthalmoplegia, and areflexia, likely secondary to vaccine-mediated immune response.

**Table 1:** Baseline investigations and CSF analysis.

Investigations	Results	Units	Reference ranges
Complete blood count			
White blood cell count	7400	/mm^3^	4000–11000
Red blood cell count	5.44	mil/mm^3^	4.5–6.0
Hemoglobin	15.9	g/dL	14.0–18.0
Hematocrit	47	%	40–50
MCV	86	fl	80–95
MCH	29	pg	27–31
MCHC	34	g/dL	32–36
Platelet count	154,000	/mm^3^	140,000–425,000
Neutrophils	81	%	50–70
Lymphocytes	12	%	25–40
Monocytes	6	%	2–10
Eosinophils	1	%	0–4
Renal function tests			
Blood urea	24	mg/dL	10–50
Blood urea nitrogen	11.2	mg/dL	8–20
Serum creatinine	0.84	mg/dL	0.7–1.2
Liver function tests			
Total bilirubin	0.9	mg/dL	0.1–1.1
ALT	22	U/L	5–55
AST	20	U/L	9–40
Alkaline phosphatase	69	U/L	30–115
Gamma GT	19	U/L	Male: < 55
			Female: <38
Cerebrospinal fluid analysis			
Appearance	Clear		Clear
CSF White blood cell count	2	cells/μL	0–5
CSF Proteins	85	mg/dL	20–40
CSF Glucose	55	mg/dL	45–80

A multidisciplinary meeting between the internal medicine, neurology, and hematology team decided to proceed with the management of MFS based on intravenous immunoglobulins, pregabalin 50 mg, and physiotherapy. The standard dosage of intravenous immunoglobulins (40 mL/kg for 5 days) was offered but was not administered due to the high costs and lack of availability. Subsequently, plasmapheresis was advised but the patient refused to undergo the invasive procedure and was discharged on request. He received regular physiotherapy sessions for the next 6 weeks. Follow-up in the outpatient clinic 6 weeks after the discharge showed that the patient's limb weakness and cranial nerve palsies had improved with complete resolution of symptoms 10 weeks after the discharge.

## 3 Discussion

The Sinovac–Coronavac vaccine is an inactivated vaccine against COVID-19, which has been approved by the World Health Organization and has an efficacy of 51% against symptomatic severe acute respiratory syndrome coronavirus 2 (SARS-CoV-2) infection and is 100% effective against severe COVID-19.^[8]^ A systematic review of central nervous system demyelination after COVID-19 vaccination showed 32 cases, out of which 5 were after Sinovac/Sinopharm vaccines.^[9]^ The majority of cases (71.8%) occurred after the first dose of the vaccine, with neurological symptoms manifesting after a median of 9 days.^[10–12]^ The pathogenesis of GBS and MFS after COVID-19 vaccination is vaguely described in the literature but is most likely due to some aberrant immune-mediated response. A proposed pathogenic mechanism is that of molecular mimicry, where a foreign antigen stimulates an abnormal immune response that targets the gangliosides of peripheral nerves.^[9]^ A brief literature review of MFS after SARS-CoV-2 infection shows several reported cases of COVID-associated MFS.^[13,14]^ Pathologically, it is plausible that SARS-CoV-2 directly induces its neuropathogenic effect on Angiotensin-converting enzyme 2 which is widely expressed in the nervous system.^[13]^ Despite its rarity, patients who present with MFS after COVID-19 vaccination usually have a favorable prognosis with remarkable recovery within a few weeks.

**Figure 1:**
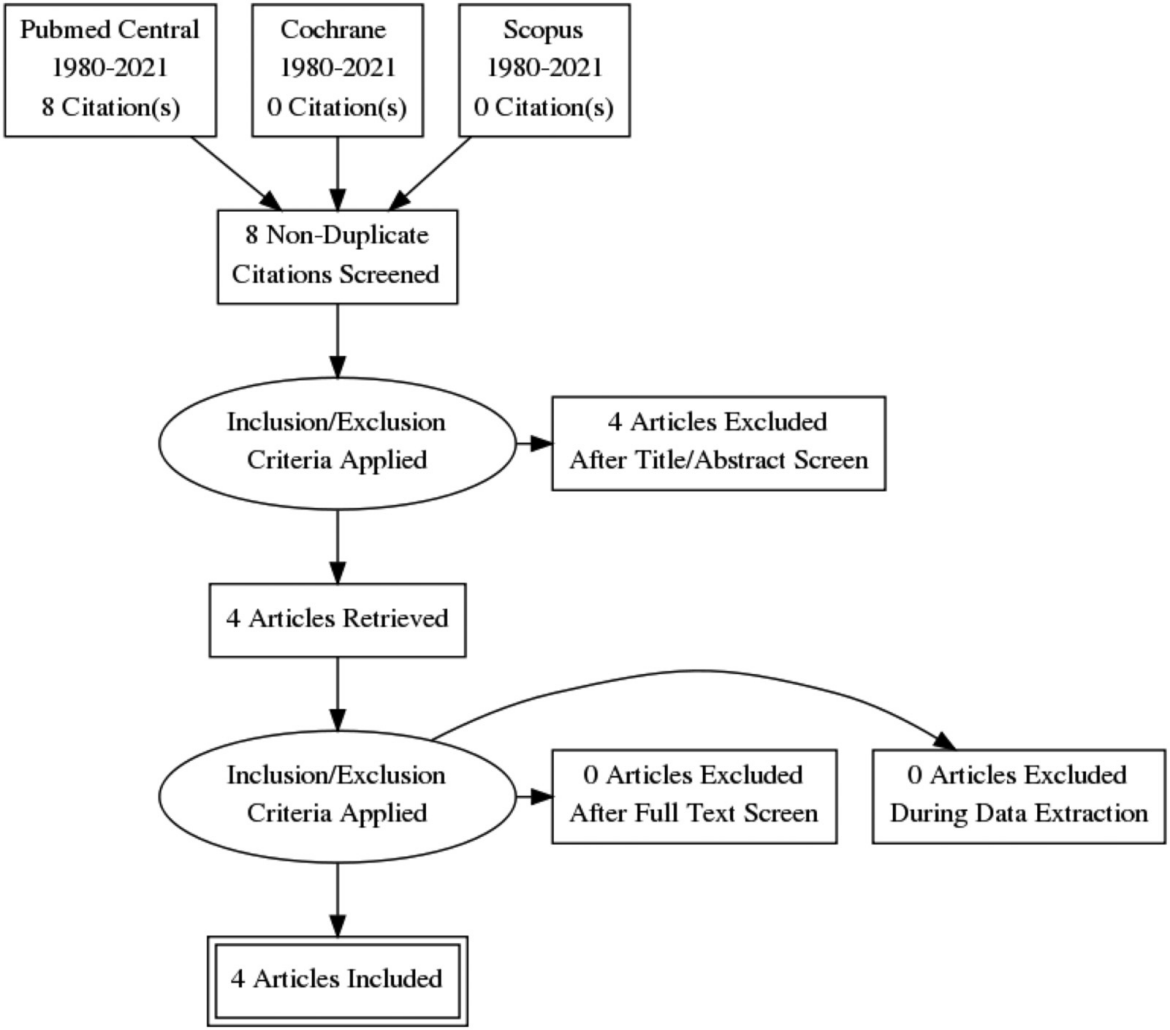
PRISMA flow diagram.

Our case describes a patient with MFS caused due to the Sinovac–Coronavac vaccination in the absence of any other identifiable triggers. Although GBS cases have been reported after influenza, measles, and meningococcal vaccines, the incidence of postvaccination GBS and its variants is infrequent, with less than one case of GBS per million immunized persons.^[15]^ Recently, a few cases of GBS have been reported after the COVID-19 vaccination.^[16]^ A literature search performed on PubMed on 03 December 2021 using Boolean operator strategy with keywords “(Miller Fisher syndrome OR MFS) AND (COVID-19 OR SARS-CoV-2) AND (vaccination)” showed 8 results (Fig. 1). The screening of abstracts revealed 4 cases reporting MFS after COVID-19 vaccination. Michaelson et al,^[17]^ Abičić et al,^[18]^ and Nishiguchi et al^[19]^ reported the neurological spectrum of MFS after Pfizer-BioNTech vaccines. At the same time, Dang and Bryson^[20]^ described an overlapping syndrome of MFS and GBS after the Oxford-Astrazeneca vaccination. The salient clinical features of these studies are mentioned in Table 2. The common feature is the presence of ophthalmoplegia and diplopia in all the reported cases. In these studies, the onset of symptoms is reportedly 14 to 18 days after the administration of COVID-19 vaccination. Nerve conduction studies were normal in most cases, and none of them showed acute neuronal changes. Among the reported cases, only Yuki and Hartung^[15]^ revealed positive antiganglioside (GQ1b) antibodies. Previous reports suggested 80% to 90% positivity of GQ1b antibodies in MFS patients.^[17,21]^ All the reported cases were managed with intravenous immunoglobulins with supportive care and showed remarkable recovery at 4 to 6-week follow-ups indicating a favorable prognosis of the COVID-19 vaccination triggered MFS.

**Table 2:** Clinical features of Miller Fisher syndrome after COVID-19 vaccination reported in the literature.

Author	Patient's age/sex	Vaccination	Clinical presentation
Nishiguchi et al^[19]^	71 years/male	Pfizer/BioNTech	Headache, oculomotor nerve palsy, ocular pain, ptosis, and limb ataxia.
Michaelson et al^[17]^	78 years/male	Pfizer/BioNTech	Mixed diplopia, paresthesia of the hands and feet, and severe gait ataxia.
Abičić et al^[18]^	24 years/female	Pfizer/BioNTech	Horizontal diplopia, impaired abduction, and elevation of eyes.
Dang and Bryson^[20]^	63 years/male	Oxford-AstraZeneca	Horizontal diplopia, bilateral facial weakness, facial diplegia, sensory ataxia, paresthesia, impaired distal lower limb proprioception, lower limb weakness, and bilateral lower limb areflexia.

A few limitations of our study should be acknowledged. Firstly, the diagnosis of MFS could not be serologically confirmed due to the unavailability of anti-GQ1b antibody testing. Secondly, we cannot exclude the possibility of an asymptomatic respiratory or gastrointestinal infection that could have been the cause of MFS.

Our case shows numerous similarities to other cases and, in addition, exhibits the classical triad of MFS along with characteristic CSF results of albuminocytological dissociation and typical electromyography findings, depicting a common pathogenic mechanism and a consistent disease course. To the best of our knowledge, this is the first reported case of MFS associated with inactivated COVID-19 vaccine. Previously, Zika virus outbreaks were also reported to be associated with GBS, with different prognoses and high mortality.^[22]^

## 4 Conclusions

MFS is a rare adverse effect after COVID-19 vaccination, and appropriate surveillance is required for early diagnosis and treatment. More than 9 billion COVID-19 vaccines have been administered worldwide and determining the causal relationship in every case of a potential adverse effect becomes challenging. Additional research is required to substantiate a temporal association between COVID-19 vaccination and MFS, and to further understand the pathophysiology behind such neurological complications, which would be vital in improving the safety of COVID-19 vaccines in the future.

## Author contributions

**Conceptualization:** Ahsun Rizwan Siddiqi, Tehrim Khan, Zohaib Yousaf

**Data curation:** Ahsun Rizwan Siddiqi, Muhammad Junaid Tahir, Muhammad Sohaib Asghar, Tehrim Khan, Zohaib Yousaf

**Formal analysis:** Ahsun Rizwan Siddiqi, Muhammad Junaid Tahir, Muhammad Sohaib Asghar, Tehrim Khan

**Investigation:** Ahsun Rizwan Siddiqi

**Methodology:** Ahsun Rizwan Siddiqi

**Validation:** Md. Saiful Islam

**Writing – original draft:** Ahsun Rizwan Siddiqi, Muhammad Junaid Tahir, Tehrim Khan

**Writing – review & editing:** Md. Saiful Islam, Muhammad Sohaib Asghar, Zohaib Yousaf

## Supplementary Material

Supplemental Digital Content
