# Supplementary material for: Miller Fisher syndrome after COVID-19 vaccination: Case report and review of literature
Source: Medicine (Baltimore). 2022 May 27;101(20):e29333. doi: 10.1097/MD.0000000000029333 (PMC9276158; doi:10.1097/MD.0000000000029333)
Supplement: Supplemental Digital Content [file medi-101-e29333-s001.docx]

**Table S1: Nerve Conduction Studies**

| Nerve tested | Distal Latencies | Amplitude |
| --- | --- | --- |
| Right median motor nerve | Normal latency | Normal amplitude |
| Right ulnar motor nerve | Prolonged latency | Normal amplitude |
| Right median sensory nerve | Prolonged latency | Normal amplitude |
| Right ulnar sensory nerve | Prolonged latency | Normal amplitude |
| Right peroneal motor nerve | Prolonged latency | Normal amplitude |
| Right tibial motor nerve | Prolonged latency | Reduced amplitude |
| Left tibial motor nerve | Prolonged latency | Reduced amplitude |
| Both sural sensory nerve | Normal response | Normal amplitude |
| Right peroneal motor nerve | Prolonged F wave latency | Reduced amplitude |
| Right tibial motor nerve | Prolonged F wave latency | Reduced amplitude |
| Left ulnar motor nerve | Prolonged F wave latency | Reduced amplitude |
| Interpretation |  |  |
| Abnormal electrophysiological study | Prolonged F wave latencies | Reduced amplitude |
| Conclusion |  |  |
| The electrophysiological study is suggestive of acute inflammatory demyelinating polyneuropathy, most probably GBS. |  |  |

**Supplemental file’s legend**

**Table S1: Nerve Conduction Studies**
